# Asylum Reform

**Published:** 1923-03

**Authors:** Barbara Ayrton Gould

**Affiliations:** Organising Secretary. National Society for Lunacy Reform, 32 & 33 Avenue Chambers, Southampton Row, W.C.


### Asylum Reform.

Sir,—The publication of the circular letter which the Board of Control in Lunacy has sent to the Clerks of the Visiting Committees of Asylums furnishes a striking instance of how authority, reluctant to admit the need of reforms, may yet be compelled by public agitation to adopt them. The reforms urged in this official circular letter are, as far as they go (which is not nearly far enough), admirable. But where do they come from?

Dr. Lomax's book, "The Experiences of an Asylum Doctor," made certain accusations against the administration of Lunatic Asylums. The usual official denials were issued. Not only that, but a Committee of Inquiry was convened by Sir Frederick Willis, Chairman of the Board of Control, to inquire into the accusations and report upon them. Before that Committee Dr. Lomax refused to appear, believing as he did that by its very constitution it was inevitably biased by the "official view." The Committee proceeded nevertheless to report. The gist of its report was that Dr. Lomax's charges were unfounded, that the agitation was uncalled for, and that apart from a few details, everything was for the best in the best of all possible asylum systems. The public remained unconvinced, and the agitation for reform continued with increasing vigour.

And now we find that the Board of Control itself has been driven to put forward suggestions, the very same that were advanced (though, of course, in slightly different words) in Dr. Lomax's book, and which would, moreover, have been almost entirely unnecessary if Dr. Lomax's accusations had not been accurate and justified. This truth, after the usual official opposition, prevails. But this is only a beginning. The virtual admission of charges which were so recently and vehemently denied should, of itself, illustrate how bad our lunacy system is, and facilitate the further meas ures of reform which are so urgently necessary.

Barbara Ayrton Gould,
Organising Secretary.

National Society for Lunacy Reform,
32 & 33 Avenue Chambers,
Southampton Row, W.C.

